# Importance of physiotherapy and occupational therapy according to people with multiple sclerosis—results from an online survey

**DOI:** 10.3389/fresc.2026.1832561

**Published:** 2026-05-12

**Authors:** Heleen Beckerman, Jacqueline P. M. A. Coppers, Isaline C. J. M. Eijssen

**Affiliations:** 1 Rehabilitation Medicine, MS Center Amsterdam, Amsterdam UMC, Location Vrije Universiteit, Amsterdam, Netherlands; 2 Amsterdam Public Health Research Institute, Social Participation and Health, Amsterdam, Netherlands

**Keywords:** allied healthcare, MS rehabilitation, multiple sclerosis, occupational therapy, patient-perceived values, physiotherapy

## Abstract

**Introduction:**

Multiple Sclerosis (MS) is a long-term neurological disease with life-altering consequences for daily functioning. While physiotherapy, occupational therapy and Cesar/Mensendieck (posture) exercise therapy are considered essential in the management of MS, little is known about how people with MS (PwMS) value these therapies across the course of the disease. Therefore, we aimed to investigate PwMS’ experiences with these therapies, as well as their perceived role and value in the lives of PwMS.

**Methods:**

A nationwide online survey was conducted among adults with MS. The survey used closed and free-text questions to assess use, experiences with these therapies, barriers to access, satisfaction, and perceived values. Quantitative data were analyzed descriptively, and free-text data were analyzed thematically.

**Results:**

In total, 193 participants (mean age 58, 73% female) completed the survey. Physiotherapy was widely known and used (97% ever used, 88% current use), occupational therapy was known to 96% and used by 75% (25% current), while posture exercise therapy was less known and used. All these therapies are highly valued by PwMS, with roles evolving throughout the disease course. Satisfaction with therapy and therapists was high, especially regarding contact, trust, and involvement in therapy decisions. Access barriers included distance, mobility, transport, and financial issues. Participants emphasized the need for MS-specific expertise of physiotherapists.

**Conclusion:**

PwMS highly value physiotherapy, occupational therapy, and posture exercise therapy, which have a lasting impact across all disease stages. Their values and needs are dynamic and multifaceted, evolving with disease progression and changing life circumstances.

## 1 Introduction

Multiple Sclerosis (MS) affects approximately 36,000 people in the Netherlands, with yearly 1,300 new diagnoses (1). Worldwide, 2.8 million people have been diagnosed with MS (2). MS leads to progressive physical, cognitive, and psychosocial challenges, impacting all aspects of daily life and living an independent life. Allied healthcare therapies, including physiotherapy, occupational therapy, and Cesar (posture) exercise therapy and Mensendieck (posture) exercise therapy, are designed to improve functions, daily activities including mobility, and societal participation. Although healthcare policymakers are advocating for evidence-based practice and value-based health care, real-world experiences and perceived values of people with MS (PwMS) regarding these therapies have not yet been investigated.

The average age at which MS is diagnosed worldwide is 32 years, and having a relatively normal life expectancy, individuals with MS may have many years to live with a disability (2). Worldwide, MS is among the top 25 of chronic health conditions and injuries contributing to a significant percentage of years of life lived with a disability that will benefit from rehabilitation (3). Because MS often starts at a young age and is chronic in nature, morbidity, expressed as years lived with disability, contributes more to the total disease burden of MS than mortality, expressed as years of life lost (4).

Advances in disease-modifying medications have significantly delayed disease progression by reducing relapses, leading to stabilized, delayed, or sometimes improved Expanded Disability Status Scale scores (5, 6). However, MS-related symptoms continue to affect many PwMS (7–10). The motor and non-motor symptoms are not only a result of the disease itself, but can also be caused by comorbidities, physical deconditioning, and aging (11–13). This co-occurrence complicates symptom management (14) and affects the quality of life of PwMS (15). In relapsing remitting MS, gait, balance, and tremor most strongly reduce quality of life, while also co-existing depression, bowel problems, spasticity, sexual dysfunction, pain, and fatigue, have a significant influence on quality of life (15). In progressive MS, spasticity, paralysis, and bowel problems are prominent and affect quality of life the most (10, 15). Furthermore, a growing proportion of PwMS report interference of MS in daily activities, from 27% in the first year to 92% after 45 years of living with MS (8).

Combining disease-modifying medication with tailored symptom management is important to lower the burden of MS (4). Early and ongoing effective MS rehabilitation can contribute to building reserve, preventing or reducing MS symptoms and limitations in daily functioning and societal participation (16, 17). Physiotherapy and occupational therapy constitute important contributions to MS rehabilitation (18) as well as posture exercise therapy, aiming to improve function, mobility, and participation. These therapies can be provided in various settings, including the community, primary care setting, inpatient clinics, or on an outpatient basis, with care differences across countries and settings (19, 20). There are no stand-alone MS institutions in the Netherlands; inpatient care is provided by specialized rehabilitation centers or general and university hospitals.

Physiotherapy is the most common allied healthcare treatment for PwMS (10, 21, 22). In the Netherlands, physiotherapy for PwMS is often provided as (self-referred) individual therapy in a primary care setting (22). Physiotherapy is also provided, specifically after the diagnosis and in complex health situations, as a short period (6 weeks) of multidisciplinary rehabilitation, mostly at outpatient hospital departments or rehabilitation centers. Physiotherapists focus on movement-related functioning, encourage healthy exercise behavior and work mostly from a biopsychosocial model, considering not only the patient's movement symptoms but also personal (e.g., health skills) and environmental factors. Patients are expected to be active partners in the treatment process through shared decision-making (23, 24). Common reasons for referring PwMS to physiotherapy are the worsening of symptoms, preventive care, the management of acute exacerbations, psychosocial issues, and palliative care (20, 25). However, there is a large diversity in physiotherapy access, service delivery, and content across Europe (20, 25, 26).

Occupational therapy also has a distinct and valuable role in MS rehabilitation (10). The occupational therapist focuses on enabling performance and engagement in meaningful activities and roles at home and in the community. Notably, physical and cognitive impairments are prominent contributors to occupational challenges in PwMS. Occupational therapy supports PwMS in maintaining independence in various areas of daily life. Therapy approaches address change at the levels of person (symptom management, coping, self-management, self-efficacy), activity (optimizing performance), and environment (adaptations, caregiver support, social networks), including e.g., training of skills, adapting activities and environments, compensatory techniques, and self-management strategies (16, 27, 28). By applying cognitive strategies, energy and time management strategies, aids and environmental adjustments, daily functioning and participation in society are optimized (29, 30).

Cesar and Mensendieck (posture) exercise therapists are also involved in MS care in the Netherlands. Cesar and Mensendieck exercise therapy are officially recognized, independent professions within Dutch allied healthcare. Both therapies aim to improve body awareness, posture, breathing, and movement. The founders, Bess Mensendieck and her former student Marie Cesar, have international roots. Between 1905 and 1924, the Mensendieck system became popular among middle and upper-classes individuals, especially in Germany, the Netherlands, Denmark, Belgium, Switzerland, France, Czechoslovakia, and the United States, where Mensendieck established several training schools (31). Nevertheless, the specific status of exercise therapist as a legally protected profession does not exist in other countries. Elsewhere, the methods are generally embedded within physiotherapy or practiced by physiotherapists specialising in this approach (31).

Given the limited research on the real-world experiences and perceived value of physiotherapy, occupational therapy, and posture exercise therapy, the aim of this study was to gain contemporary insight into how people with multiple sclerosis use and experience these therapies and to understand the perceived values, roles, benefits and barriers associated with these therapies across the course of their disease.

## 2 Methods

### 2.1 Study design and participants

A cross-sectional online survey was conducted from October 2024 to April 2025, aiming to include adults (≥18 years) from across the Netherlands with a confirmed MS diagnosis who were proficient in Dutch. No additional inclusion or exclusion criteria were applied. PwMS were recruited via media channels including digital newsletters, website posts, social media, and patient and professional networks in the Netherlands. In addition, paper flyers were distributed at outpatient clinics to reach people who are less familiar with digital media. Interested individuals were invited to email the research team, and those who responded received a personalized email containing information about the study and a unique link to the survey. One participant was unable to complete the survey independently and was therefore interviewed by telephone by a member of the research team.

The Medical Ethical Committee of Amsterdam UMC (2024.0646) determined that this research was not subject to the Dutch Medical Research involving Human Subjects Act. Written informed consent was obtained online from each participant before the online survey participation.

### 2.2 Survey

The online survey started with questions about the awareness and use of physiotherapy, occupational therapy, and Cesar and Mensendieck exercise therapy, followed by sections on demographics, health status, current and past experiences with these therapies. At the beginning of the survey, Cesar and Mensendieck (posture) exercise therapies were combined. Therefore, we most often refer to posture exercise therapy throughout the remainder of this article. Among users, key details about the consultation aim, content, duration, perceived effects of three therapy episodes per type of therapy, satisfaction and barriers were requested. A top three of perceived therapy effects could be selected from a list of 16 topics. For satisfaction and barriers, predefined lists were also utilized.

For assessing the actual health status of participants, the following patient-reported outcome measures were used: MS Walking Scale-12 (MSWS-12), MS Impact Scale (MSIS-29), and the 10-item short form Arm Function in Multiple Sclerosis Questionnaire (AMSQ-SF). Scores are expressed on a 0-100 scale, with higher scores indicating higher impact of MS. MSWS-12 contains 12 questions regarding walking abilities (32). MSIS-29 assesses the impact of MS on daily life activities (20 physical health and 9 psychological health questions) (33). The AMSQ-SF provides an efficient way to measure arm and hand function in MS patients (34, 35). As part of the questions about future expectations, the Exercise Self-Efficacy Scale (ESES) was used to assess participants' self-confidence in staying physically active. It consists of 10 questions, with 100 indicating very high and 0 indicating very little self-confidence (36).

The survey was designed and disseminated using Survalyzer, an online survey system, and consisted of closed and free-text questions. In case the survey was not (fully) completed, a personal reminder e-mail was sent approximately 6 weeks later. A new survey was sent upon request.

### 2.3 Data analysis

#### 2.3.1 Quantitative data

Quantitative data were analyzed descriptively, using SPSS version 28. For the satisfaction scores, the 5-point Likert scale was dichotomized into (very) satisfied vs. neutral and (very) dissatisfied.

To clarify how patient needs, therapy content, and the perceived roles of physiotherapy and occupational therapy changed over time and across MS disease stages, we conducted additional analyses of therapy episodes. For these analyses, we took into account participants' age, disease duration, the calendar years in which the therapy episodes took place, and free-text information per episode about patients' reported reasons for initiating therapy and perceived effects.

Following the example of disability severity classifications based on the Expanded Disability Status Scale, we distinguished five clinically relevant disease stages: (1) immediately after diagnosis, (2) mild MS: minimal noticeable movement difficulties, (3) moderate MS: movement, walking, and balance become more challenging, daily functioning requires more physical and cognitive effort, (4) advanced MS: assistive devices are needed both indoors and outdoors (such as a wheelchair), and support from informal caregivers becomes more frequent, (5) proactive palliative stage in the final years with MS. Cesar/Mensendieck exercise therapy was excluded from this analysis due to the limited number of therapy episodes reported. For descriptive purposes, therapy episodes were subsequently grouped into these five stages based on the combination of self-reported disability indicators (e.g., walking ability, assistive device use), disease duration, and age, as well as the narrative information provided in the free-text descriptions.

#### 2.3.2 Free-text questions and thematic analysis

For each therapy, one open-ended question was included to explore patient-perceived roles and values. For physiotherapy, the question was: “What does physiotherapy mean to people with MS like you? In your own words, can you describe what physiotherapy means to you? How has physiotherapy affected your daily life?” The same question was asked for occupational therapy and for posture exercise therapy, by substituting the name of the therapy.

All free-text responses were pooled per therapy and analyzed thematically. An inductive thematic analysis was performed to identify recurring themes and patient-perceived “key values” of each therapy. The thematic analysis was supported by a secure AI-based large language model tool developed by Amsterdam UMC; the researchers remained fully responsible for checking, refining, and interpreting the themes and their descriptions.

## 3 Results

### 3.1 Participants

In total, 193 PwMS participated, of whom 179 (92.7%) fully completed the survey. The participants' postal codes showed a representative distribution across the Netherlands. Mean age was 58 years (SD 10.4), 73% female, 24% employed, 51% on disability pension (Table 1). The duration of MS ranged from 0.5 to 61 years, with 20.7% of the participants having MS for five years or less, and 33.7% having MS for more than 20 years (Table 1). More than 40% had a comorbidity for which they consulted a medical specialist (Supplementary Table S1). Most participants reported a substantial burden from MS, with correlations up to 0.73 between their perceived burden and scores on MSIS29, MSWS12, and AMSQ-SF (Supplementary Table S2).

**Table 1:** Sociodemographic and disease-related characteristics of the participants (*n* = 193).

Sociodemographic characteristic	N	%
Gender		
Female	141	73.0
Male	47	24.4
Other preference	5	2.6
Age (yr) mean, sd	58.0	10.4
Age (yr)		
<40	11	5.7
40–49	22	11.4
50–59	56	29.0
60–69	72	37.3
70>	18	9.3
Missing	14	7.3
Educational level		
Low	7	3.6
Medium	63	32.6
High	106	54.9
Missing	17	8.9
Living situation		
With partner	138	77.1
With children	48	26.8
Institution	3	1.7
Employment (yes)	43	24.0
Fulltime (≥36 h/week)	12	6.7
Parttime (<36 h/week)	31	17.3
Social benefits		
Disability pension	98	50.8
Sick leave (max 2 years)	5	2.6
(Early) retirement	19	9.8
No	56	29.0
Missing	15	7.8
Unpaid work		
Volunteer work	81	45.3
Informal caregiver	19	10.6
MS characteristics		
Duration MS (yr) (median, IQR)	15	[6.5–25]
Duration MS (yr)		
0–5	40	20.7
6–10	35	18.1
11–15	27	14.0
16–20	26	13.5
21–30	44	22.8
>30	21	10.9
Comorbidities		
No	112	58.0
Yes	81	42.0
Able to walk		
Yes	175	90.7
No	15	7.8
Missing	3	1.6
Perceived Burden MS		
No	1	0.5
Little	22	11.4
Moderate	94	48.7
Much	65	33.7
Very much	11	5.7
MSIS29 physical [0–100]	36.9	23.8–53.8
MSIS29 psychosocial [0–100]	22.2	11.1–38.9
MSIS29 total [0–100]	33.6	22.2–47.4
MSWS-12 [0–100]	60.4	29.2–81.3
AMSQ-SF [0–100]	8.0	0.0–24.0
ESES [0–100]	80.0	66.7–86.7

AMSQ-SF, 10-item short-form Arm Function in Multiple Sclerosis Questionnaire; ESES, Exercise Self-Efficacy Scale; IQR, Interquartile range; MSIS29, MS Impact Scale; MSWS-12, MS Walking Scale-12.

### 3.2 Therapy awareness and utilization

Table 2 provides data on therapy awareness, and current and past use of therapies. All participants had heard of physiotherapy, and 96% were aware of occupational therapy. Cesar and Mensendieck exercise therapy were unknown to 27%-28% of participants (Table 2).

**Table 2:** Therapy awareness and utilization of allied healthcare.

Type of therapy	Ever heard of (%)	Ever used (%)	Currently using (%)
Physiotherapy	100.0	96.8	88.4
Occupational Therapy	96.3	75.4	25.3
Cesar Exercise Therapy	71.6	11.4	2.6*
Mensendieck Exercise Therapy	73.2	19.0	2.6*

* 2.6% currently use Cesar or Mensendieck exercise therapy. This was not requested for Cesar and Mensendieck therapy separately.

Of the current physiotherapy users (*n* = 168), most received physiotherapy for more than a year (88.1%), often weekly (27.4%) or two or more physiotherapy sessions per week (51.8%). Physiotherapy takes place at a local primary care practice and mainly consists of individual therapy sessions. Users expect to need physiotherapy for a longer period (Supplementary Table 3).

Occupational therapy was received more episodic, tailored to specific needs. For 25.6% of the current users (*n* = 43) the frequency of occupational therapy sessions was determined by their needs and varied over time. The current therapy location was a primary care practice in the neighborhood (26.8%), a rehabilitation department (29.3%), or at home (31.7%). Over 65% believe that their current therapy period can be completed after some time (Supplementary Table S3).

Regarding posture exercise therapy, the four current users received therapy for over a year, with a frequency of one session per week (50%) or one or two sessions per month (50%) (Supplementary Table 3).

### 3.3 Barriers to access

A small percentage of participants experience barriers in consulting a therapist; 14.2% of the participants experienced barriers consulting a physiotherapist, and smaller percentages experienced barriers to consulting an occupational therapist (8.1%) or exercise therapist (5.3%) (Table 3). Main barriers included distance to specialized practices, mobility and transportation issues, and financial constraints.

**Table 3:** Perceived barriers.

Perceived barriers	Physiotherapy *N* = 183 (%)	Occupational Therapy *N* = 173 (%)	Posture Exercise Therapy *N* = 152 (%)
No	157 (85.8)	159 (91.9)	144 (94.7)
Yes (any barrier)	26 (14.2)	14 (8.1)	8 (5.3)
Type of perceived barriera^a^			
No referral from GP or physician	1 (0.5)	2 (1.2)	2 (1.3)
Distance to practice	11 (6.0)	4 (2.3)	1 (0.7)
Financial (costs/unclear)	6 (3.2)	-	2 (1.3)
Transportation issues	4 (2.2)	1 (0.6)	4 (2.6)
Wait list	3 (1.6)	2 (1.2)	-
Quality therapy/therapist	2 (1.1)	3 (1.7)	1 (0.7)
Personal reason	1 (0.5)	2 (1.2)	-

a Multiple barriers could be checked.

### 3.4 Satisfaction

Table 4 shows that PwMS are (very) satisfied about most aspects of the therapy and the therapist. The full data are presented in Supplementary Table S4 and show that more than 63% of current physiotherapy users are *very* satisfied with their contact with the physiotherapist, the therapy they receive, their trust in the physiotherapist, and their active involvement in the therapy. A small percentage is dissatisfied with the physiotherapist's knowledge of MS (4.8%), the physiotherapist's collaboration with other healthcare providers (5.4%), and the cost of physiotherapy (8.4%) (Supplementary Table S4).

**Table 4:** Percentages of participants who are satisfied or very satisfied with physiotherapy, occupational therapy and posture exercise therapy.

Satisfied about	Physiotherapy *N* = 168 (%)	Occupational Therapy *N* = 43 (%)	Posture Exercise Therapy *N* = 4 (%)
a. The therapy you are receiving or have received in the past 3 months	92.9	93.0	100
b. Your therapist's knowledge of MS	82.1	86.1	100
c. How therapy matches your request for therapy	88.0	90.7	100
d. How therapy fits your personal situation	90.5	93.0	100
e. The results of the treatment (so far)	82.1	83.7	100
f. The trust you have in your therapist	94.1	88.4	100
g. The time available for a therapy appointment	86.3	93.1	100
h. The contact you have with your therapist	94.1	93.0	100
i. The input you have in the therapy you receive	90.5	90.7	100
j. The therapy aligns with your needs and preferences	88.7	88.3	100
k. Availability of therapy at a time that suits you and as quickly as you want	84.0	86.0	100
l. How your therapist collaborates with other healthcare providers	64.0	79.1	100
m. The accessibility of the therapy practice	91.1	83.7	100
n. The amount you have to pay for the therapy yourself	63.7	51.2	100

Regarding occupational therapy, users are particularly satisfied with their trust in the occupational therapist, their contact with the therapist, and the possibility to express their needs (Table 4 and Supplementary Table S4). Only one or two people (≤ 4.7%) were dissatisfied with the contact, the alignment of occupational therapy to their needs and preferences, the availability of occupational therapy, collaboration with other healthcare providers, the accessibility of the practice, and the cost they had to pay for occupational therapy themselves.

The four responders were (very) satisfied with Cesar or Mensendieck exercise therapy (Table 4, Supplementary Table S4).

### 3.5 Thematic analysis of perceived roles and values of therapies

In their own words, participants described the significance and impact of physiotherapy, occupational therapy, and posture exercise therapy on their lives. For each therapy, responses to one open-ended question were pooled and analyzed thematically to identify key values. The resulting themes are summarized in Table 5.

**Table 5:** Patient-perceived role and value of physiotherapy, occupational therapy and posture exercise therapy in the lives of people with MS, based on thematic analysis of responses to a single open-ended question per therapy.

Key values physiotherapy	Explanation by people with MS
Maintaining physical function and mobility	Physiotherapy is crucial for preserving muscle strength, balance, coordination, and mobility. It helps to slow down MS progression and to prevent physical deterioration.
Pain relief and reduction of muscle stiffness	Participants frequently cite physiotherapy as effective for alleviating pain and reducing muscle stiffness.
Psychological and mental benefits	Physiotherapy offers mental support, boosting self-confidence, improving mental health, and aiding coping strategies. The social aspect of group sessions adds to these benefits.
Enhanced motivation and structure to remain active	Regular physiotherapy appointments provide structure and motivate participants to remain active.
Personalized guidance	Participants appreciate tailored support from physiotherapists, who adapt exercises to their individual needs and limitations. MS-specific expertise is considered essential. While training requires energy, physiotherapists provide energy, encouragement and support.
Recovery after relapse	Physiotherapy is important for regaining muscle strength, balance, and mobility following a relapse or setback.
Prevention and maintaining current physical functioning	Physiotherapy is seen as a preventive measure to maintain current physical functioning. It gives people a sense of control.
Social interaction and peer support	Physiotherapy offers opportunities for social engagement and peer support, helping participants share experiences and gain perspective.
Self-reliance and home exercises	Participants learn exercises they can perform independently, increasing self-reliance and control over their health.
Improved quality of life & well-being	Many participants consider physiotherapy essential for enhancing physical, mental, and psychosocial well-being.

Key values occupational therapy	Explanation by people with MS
Energy and time management, boundary setting, and acceptance	Occupational therapy helps PwMS to manage their energy by conscious planning of daily activities, pacing activities, and distributing energy throughout the day or week. It also fosters insight into personal resilience, boundary setting, and acceptance of limitations, leading to adaptive coping with the disease.
Self-reliance, independence, and practical support	Participants value occupational therapy for promoting independence, teaching new strategies, and providing practical tips for daily living. The therapy offers concrete advice to simplify everyday activities.
Advice and support with assistive devices and environmental adaptations (e.g., home, workplace)	Occupational therapists play a key role in advising, selecting, and facilitating the use of assistive devices (e.g., wheelchairs, shower chairs, grab rails, adapted bicycles) and implementing home adaptations. They also assist with applications to agencies such as the Social Support Act (WMO) and related administrative processes.
Acquiring cognitive skills and psychological support	Participants report that occupational therapy addressed cognitive issues, such as concentration and memory, by teaching skills. Occupational therapy also provides psychological support and a listening ear, helping individuals cope with the emotional impact of MS.
Enhancing social participation	Occupational therapy enhances social participation by addressing practical and physical limitations. Assistive devices provided by occupational therapists significantly support continued engagement in society.
Long-term impact and benefits	PwMS note the lasting benefits of occupational therapy. They continue to apply the insights, skills, and tools acquired, regularly revisiting principles of energy and time management to better organize their daily activities. Learned skills and assistive devices support ongoing independence and self-reliance, while fostering a better understanding and management of MS.

Key values posture exercise therapy	Explanation by people with MS
Enhanced body and movement awareness	Exercise therapy increases participants’ awareness of their physical capabilities and limitations, helping them improve movement patterns and posture.
Improved posture in daily activities	Participants view exercise therapy as a means to develop better posture, which can alleviate symptoms and promote more efficient energy management.
Positive effects on balance and stability.	Exercise therapy contributes to improved balance and stability, which is crucial for managing MS progression.
Valuable complement to physiotherapy	Some participants regard Cesar/Mensendieck exercise therapy as a valuable complement to physiotherapy, offering a distinct approach and empowering them to manage their symptoms independently.

#### 3.5.1 Importance of physiotherapy

Importance of the responses of 177 PwMS indicate that physiotherapy has a multidimensional impact on PwMS. Key values of physiotherapy are described in Table 5. Physiotherapy provides physical, mental, and psychosocial benefits, maintains physical function and mobility, motivates people to stay active, helps prevent further deterioration, and is regarded as essential for enhancing self-reliance and quality of life. Negative experiences concerned limited access, insufficient MS-specific knowledge among therapists, symptom worsening, therapy demands, and disappointment with outcomes in advanced stages of MS.

Participants emphasized that physiotherapists treating PwMS must possess specialized knowledge of MS, and skills to understand the complexity and fluctuating symptoms of MS, and to develop individualized treatment plans that adapt to daily variations, co-occurring symptoms, and the disease stage. Expertise in addressing MS-specific issues, such as spasms, fatigue, balance problems, and muscle weakness, is considered essential, as is experience with targeted interventions like neurorehabilitation, balance training, and spasm management. Participants value physiotherapists who recognize and address the physical and psychosocial impact of MS, who communicate effectively and collaborate with other healthcare providers, such as occupational therapists, rehabilitation physicians and neurologists. The ability to promptly identify (unusual) changes in health status and refer patients to other professionals or complementary treatments is highly regarded, as well as flexible and accessible physiotherapy services, particularly during acute symptoms or relapses.

#### 3.5.2 Importance of occupational therapy

Importance of occupational therapy involved occasional or short-term consultations, or more intensive, multidisciplinary rehabilitation programs lasting 6-12 weeks. According to the single open-question responses of 130 PwMS, occupational therapy helps them to manage their energy by conscious planning of daily activities, pacing, and distributing energy throughout the day or week. It also fosters insight into personal resilience, boundary setting, and acceptance of limitations, leading to adaptive coping with MS. Other key values of occupational therapy are summarized in Table 5.

Participants did not explicitly mention the need for MS-specific knowledge among occupational therapists. However, they value effective symptom management, up-to-date knowledge of assistive devices, and appropriate provision of such devices by their therapists.

#### 3.5.3 Cesar and Mensendieck exercise therapy

From the free text responses of 46 participants with experience in Cesar and Mensendieck exercise therapy, four key values were identified, namely enhanced body and movement awareness, improved posture in daily activities, positive effects on balance and stability, and it is a valuable complement to physiotherapy (Table 5). Participants did not mention the need for MS-specific knowledge among posture exercise therapists.

### 3.6 Value of physiotherapy and occupational therapy in different MS disease stages

Table 6 presents the perceived patient values over time, with in rows the characteristics of physiotherapy, the physiotherapist, and participant quotes about physiotherapy that illustrate perceived changes over time. The columns represent the disease stages we distinguished. The selected quotes are representative and reflect the experiences of the participants. The same is shown for occupational therapy (Table 7).

**Table 6:** Role and value of physiotherapy and physiotherapists in different MS disease stages according to people with MS (thematic analysis).

Characteristic	Immediately after diagnosis	Mild MS	Moderate MS	Advanced MS	Proactive palliative stage
Physiotherapy	- Explanation, basic movement, fitness, strength, balance - Learning to cope with the diagnosis - Building trust	- Recovery after relapses/progression - Building an active lifestyle - Learning to manage recurring symptoms	- Maintaining mobility, balance, strength - Reducing muscle stiffness/pain - Support with assistive devices - Optimizing daily functioning	- Preserving remaining functions - Preventing complications - Pain relief - Comfort and transfers	- Comfort, pain relief - Preventing complications - Quality of life - Support with loss of functions
Physiotherapist	- Guide, reassurance, motivator - Provides explanations - Listening ear - Helps with acceptance	- Expert guide - Motivates, monitors boundaries - Personal attention - Detects changes	- Motivator, coach, accountability - Provides tailored care - Attention to psychosocial and social aspects	- Steady support - Motivates, thinks along - Alert to changes - Adjusts treatment	- Steady support, listening ear - Comfort and trust - Helps cope with loss of functions - Psychosocial support
Participants’ quotes about physiotherapy	“The physiotherapist showed me what is possible with MS.” “I appreciate the guidance, as it is sometimes difficult to determine my limits."	“The physiotherapist encourages me, always comes up with useful and fun exercises. He cheers me up, gives good advice, and listens."	“The physiotherapist gives me energy, even though training actually costs energy. It motivates me to keep training after all these years.” “The physiotherapist tailors the treatment to my personal situation, abilities, and limits."	“The physiotherapist is an important part of my life. If my therapist is on leave, I sometimes skip a week. By the end of the week, I immediately have pain in my arms, legs, shoulders, and back.” “The physiotherapist notices when I am doing worse and adjusts the exercises, and also sees when I am doing well and then we try to do more."	“Physiotherapy has become an important part of my life. Because she comes weekly, I can enjoy the things I want to do more. With pain, everything is harder.” “The physiotherapist is a great support, explains what is happening and what she can do, but also makes it clear sometimes it is just part of life”.

**Table 7:** Role and value of occupational therapy and occupational therapists in different MS disease stages according to people with MS (thematic analysis).

Characteristic	Immediately after diagnosis	Mild MS	Moderate MS	Advanced MS	Proactive palliative phase
Occupational Therapy	- Practical advice for daily life - Energy balance, daily planning - Coping with cognitive symptoms	- Learning to pace, energy management - Practical advice on daily functioning - Advice on assistive devices	- Solutions for daily problems - Applying for/using assistive devices - Advising/arranging home adaptations - Energy balance	- Advising/arranging home adaptations - Applying for assistive devices - Maintaining independence	- Maximizing comfort - Adapting the environment - Advice on assistive devices - Support for informal caregivers
Occupational Therapist	- Coach, provides insight - Thinks along - Helps with planning/structuring - Support with acceptance	- Advisor, finds solutions together	- Coach who helps with reflection	- Expert with practical tips - Supports with assistive devices - Listening ear	- Practical supporter, point of contact, coordinator - Helps with applications for facilities - Provides practical and emotional support
Participants’ quotes about occupational therapy	“The occupational therapist is helpful because she thinks along and has solutions or advice for things I encounter.” “It has given me more control over my energy expenditure.” “I have learned to plan my day, ensuring a proper balance between rest and activities."	“The occupational therapist checks in when I get stuck. If I need a new assistive device, she advises me and helps with adjustments if needed."	“The occupational therapist is a point of contact and support for all kinds of problems in daily life, big or small."	“The occupational therapist takes a lot of administrative work off my hands and has direct lines with agencies.” “The occupational therapist looks for what is best for you, not what is most expensive."	“The occupational therapist ensures you can keep going. I think every MS patient needs that. For me, it is a huge support.” “The occupational therapist is a source of information and support for all kinds of problems in daily life, both big and small.” “She gave me tools to deal with my poor memory and ultimately arranged a comfortable wheelchair for me."

## 4 Discussion

This survey study highlights the indispensable role of physiotherapy and occupational therapy in the lives of PwMS in the Netherlands. Physiotherapy is valued for supporting mobility, physical fitness, and maintaining independence. The therapeutic environment in physiotherapy and the continuous guidance of experienced physiotherapists are especially appreciated for continued coping with the progressive nature of MS. The role of physiotherapy evolves with disease progression, requiring individualized and adaptive approaches to optimize functioning. The perceived importance of physiotherapy for participants' future functioning, along with their sense of self-efficacy, underscores the necessity for continuous and adaptable support from physiotherapists. The limited use and awareness of posture exercise therapy suggest opportunities for further patient education and integration.

Occupational therapy is highly valued for the support it provides with coping with daily challenges, energy and time management, acquiring cognitive skills, psychological support, enhancing independence, and social participation in meaningful activities. Its involvement in advising on and enabling assistive devices and environmental modifications is essential, considering both the individualized needs and the often-complicated application procedures for assistive devices. Occupational therapy has a long-lasting impact, underscoring the value of this therapy as a sustainable healthcare facility for PwMS. The evolving occupational therapy-related needs in daily functioning during the MS lifespan call for flexible access and patient-centered therapy approaches. The perceived benefits are consistent with the findings of a recent Cochrane review (37), which showed that occupational therapy may improve daily functioning and mental health-related quality of life for PwMS. Our study results are also in line with similar findings of a meta-synthesis of cognitive rehabilitation by occupational therapists (38) and add to the limited evidence base from occupational therapy trials in MS to date (28–30, 39).

In addition to this survey study, we interviewed 23 PwMS individually (40). In-depth findings of the interview study and the survey study revealed that PwMS place high value on continuous physiotherapy and occupational therapy, especially when tailored to their personal needs and disease progression. Five interrelated key themes emerged from the interviews: values related to the patient themselves, the therapist, the patient-therapist relationship, the therapy, and collaboration among healthcare providers. More importantly, we observed a growing preference among PwMS for self-determination, self-management, and involvement in decision making over the lifespan.

Patient values are an important pillar of evidence-based medicine (EBM), next to clinical expertise of the therapist and scientific literature. Therefore, evidence-based MS care requires at least understanding of patient values, needs, preferences, and priorities over their lifespan (41). With the growing shift towards value-based MS healthcare and shared decision making, personal values of PwMS should be considered (42). A careful dialogue between therapist and patient, together with an integrated registration of patient-relevant information covering symptoms, daily functioning, societal participation, and wellbeing, contribute to improved healthcare outcomes in areas that matter the most to PwMS (43, 44). With the intention of optimizing patients' daily functioning and enabling meaningful participation, health professionals need to pay attention to the importance of contextual (personal and environmental) factors. This is highlighted by the International Classification of Functioning framework that clarifies the dynamic interaction between a person's health condition, environmental factors and personal factors (45, 46). Physiotherapy and occupational therapy registries that systematic record therapy characteristics and patient-relevant outcomes can provide real-world insight into the clinical relevance and long-term effectiveness of these allied healthcare therapies (10, 47, 48).

According to participants in our study, physiotherapy serves an important preventive measure. They continuously strive to delay decline, thereby preventing and/or postponing additional care needs. It gives PwMS a sense of control and self-efficacy in their efforts to prevent further health decline. Participants reported that even a temporary interruption of physiotherapy leads to a noticeable decline in physical fitness, motivating them to continue therapy. These findings are in line with Roikjaer et al., who found that physical activity is a pathway to existential growth and improved wellbeing that goes beyond traditional health outcomes in MS and other chronic diseases (49).

### 4.1 Early induced brain-healthy lifestyle

Physiotherapy serves as an effective symptomatic treatment for PwMS, and growing evidence highlights the impact of physical activity and exercise in modifying disease progression and reducing etiological risk factors associated with the development of MS (50). Therefore, Dalgas and colleagues et al. introduced the exercise-induced postponement theory and urged for a paradigm shift in which tailored exercises are prescribed synergistically with disease-modifying medication as “medicine” to each person with MS at the earliest window of opportunity (50, 51). Early-phase exercise is already standard in Denmark by offering PwMS free-of-charge physiotherapy (52). Similarly, the National MS Society in the United States recommends regular and ongoing physiotherapy for PwMS, starting at the point of diagnosis (53). The benefits of a (brain-)healthy lifestyle, including habitual physical activity and exercise therapy are well established (54–56). MS neurologists can have a powerful role in promoting healthy exercise behavior and referring to physiotherapy from diagnosis onward (41, 57–59).

Maintaining good health and a (brain-)healthy lifestyle requires continuous attention and physical and mental resilience (60). Participants in our study recognize the importance of a healthy and active lifestyle, and a positive mindset. Regular physiotherapy and occupational therapy contribute to keeping PwMS as active as possible and help prevent dependence, loss of participation in society, and loss of autonomy (21).

### 4.2 Provision of physiotherapy and occupational therapy from an international perspective

The provision and reimbursement of physiotherapy and occupational therapy in PwMS varies largely between countries (20, 25, 26, 61). In the Netherlands, a maximum of 10 h of occupational therapy are reimbursed annually and physiotherapy is generally not covered by the basic health insurance, unless part of multidisciplinary rehabilitation. For some chronic conditions, such as MS, only the first 20 physiotherapy sessions are not covered, but subsequent sessions are reimbursed if medically indicated. Citizens can purchase additional health insurance or pay for these therapies themselves. Some study participants reported financial barriers to join therapy. Denmark provides free of charge physiotherapy. Research showed that free of charge physiotherapy started in 13% of PwMS within 2 months of their MS diagnosis, in 49% within the first 2 years after diagnosis and 61% at 5 years from diagnosis (52). These percentages may be underestimated, as some Danish PwMS may have received municipal physiotherapy. Australia facilitates early access of physiotherapy at the point of diagnosis (62).

Patients attending a public Australian hospital are expected to be provided with information, as well as access to treatment and specialized care during their initial appointment. In a survey of 160 PwMS, 52% indicated that they had consulted either a hospital-based or private physiotherapist (62). In Switzerland, physiotherapy is reimbursed by mandatory health insurance if it is prescribed by a physician. Prescriptions are usually limited to nine sessions after which new prescriptions are necessary up to a total of 36 sessions yearly. Severely affected patients may receive cost approval by the health insurance for long-term physiotherapy over many years (48). In Switzerland and the Netherlands out-of-pocket expenditures such as insurance deductibles can be prohibitive for accessing occupational therapy or physiotherapy. In Italy, short 4-week therapy periods from physiotherapists with experience in neurological MS rehabilitation are usually prescribed, with 2-3 sessions of 45 min per week (63, 64). Inpatients receive a minimum of 2 sessions per day, 5 times per week (63, 64). In a recent study from France, Bonnyaud et al., compared 4-week outpatient interdisciplinary specialized MS rehabilitation vs. 4-week general physiotherapy, each with 4 sessions per week (65). Based on their findings, the authors proposed an annual 4-week intensive rehabilitation program in MS clinics, in addition to (non-specialized) community physiotherapy provided throughout the year (65).

These international differences in healthcare, together with political and financial factors, create disparities in access to evidence-based care. The MS Barometer 2020 of the European MS platform (61) confirms that such variation in health system organization and service provision is a major barrier to optimal MS rehabilitation across Europe. Expanding the EBM model to include health system and organizational factors is therefore crucial to improve equitable access for PwMS (66). To improve access to physiotherapy and occupational therapy in the Netherlands, Dutch healthcare policymakers are advised to expand reimbursements and simplify procedures.

### 4.3 Specialized MS knowledge and expertise of therapists

Similar to previous studies (41, 57, 67), our participants emphasized the importance of specialized MS knowledge among physiotherapists. Specialized MS knowledge of therapists enables continuous personalization and timely adjustment of therapy. Specialized physiotherapy for people with Parkinson's disease might serve as a model, as the ParkinsonNet approach is associated with enhanced outcomes, fewer complications and lower healthcare costs, as compared to usual physiotherapy (68, 69).

### 4.4 Integrated MS care

In the absence of a cure for MS, physiotherapy and occupational therapy should be integral components of care throughout the disease course, supporting optimal long-term functioning through specialized early intervention, rehabilitation, and patient education. Optimizing functioning is the objective of rehabilitation (3). According to the World Health Organization (WHO), functioning is essential to a person's wellbeing and is therefore recognized as the WHO's third health indicator, alongside mortality and morbidity (70). In an ageing world where people are living longer—often with increasing limitations—advocating for optimal functioning is more urgent than ever, including for people with MS (19, 71).

Despite these needs, the current focus on brain health and minimizing MS disease activity has led to less attention for treating patient-relevant symptoms that impact daily life (72, 73). The effective treatment of focal neuroinflammation with an increasing number of disease-modifying medications has delayed the onset of motor symptoms, making less visible symptoms more prominent. A lack of awareness regarding (invisible) MS symptoms and their non-pharmacological treatment options may result in many patients with fatigue, spasticity, cognitive dysfunction, bladder and bowel problems, or sexual dysfunction being sub-optimally treated or untreated (10, 74, 75).

While this study focused solely on patient values regarding physiotherapy, occupational therapy, and posture exercise therapy, a complex disease such as MS requires a comprehensive, interdisciplinary approach. The 2023 Impact of Multiple Sclerosis Symptoms (IMMS) survey among more than 17,000 European PwMS revealed that, on average, individuals experience 13-14 symptoms, of which less than 37% are well managed. The proportion of untreated symptoms ranged from 73% for speech and swallowing difficulties to 17% for muscle weakness and 7% for mobility impairments (74). Improved symptomatic treatment is essential for enhancing the wellbeing of PwMS and reducing their disease burden.

To achieve optimal MS care, international interdisciplinary standards are needed to facilitate close collaboration among healthcare professionals, with mutual recognition and respect for each discipline's strengths. The preferred standard of care is a multidisciplinary MS rehabilitation team—including a rehabilitation physician, physiotherapist, occupational therapist, neuropsychologist, clinical psychologist, social worker, and sexologist—focused on optimizing patients' daily functioning (3, 76). Timely involvement of other specialists, such as speech therapists and dietitians, is also recommended (21). Close collaboration with MS-specialized neurologists and nurses regarding disease-modifying medication plans, as well as with other physicians such as internists, urologists, or geriatricians, ensures a comprehensive approach to MS care by integrating expertise on MS-specific symptoms, comorbidities, and age-related health issues (77). International standardization of symptom management and MS rehabilitation is urgently needed (61, 75, 78).

### 4.5 Strengths and limitations

A strength of our study is the inclusion of PwMS from across the Netherlands and from the entire disease spectrum, from those recently diagnosed to individuals with long-term MS. The comprehensive evaluation of patient satisfaction and perceived value across physiotherapy, occupational therapy, and posture exercise therapy for people with MS, using both quantitative and qualitative data, together with detailed patient perspectives on therapy roles and benefits at different disease stages, provides valuable insights into the role of allied healthcare in people's lives.

However, a potential limitation is that our recruitment methods may have attracted highly educated participants who were more likely to use online platforms and were already familiar with physiotherapy and occupational therapy. Furthermore, the use of an online survey may have excluded individuals with cognitive impairments, lower health or technology literacy, lower socio-economic status, or different native languages (79).

Furthermore, we did not collect a direct patient-reported disease stage (e.g., using the Patient Determined Disease Steps) for each therapy episode. Consequently, the five MS disease stages used in our descriptive analysis were not based on a formal, episode-specific disability measure, but on a pragmatic grouping informed by disease duration, age, and patients' narrative descriptions of reasons for starting therapy and perceived effects. This pragmatic staging may have introduced some degree of misclassification, and the patterns described across disease stages should therefore be interpreted as indicative rather than definitive.

In our study, 97% and 75% of participants had previous experience with physiotherapy and occupational therapy, and 88% and 25% were current users, respectively. A comparable Australian survey found that 52% of 160 respondents had seen a physiotherapist (62). In the German MS registry, 84% of PwMS reported using physiotherapy and 43% occupational therapy (10), while in the Swiss registry, these figures were 44% and 5%, respectively (80). Future research should explore the perspectives of underrepresented groups and whether socio-economic and cultural factors cause inequalities in access to and experiences with physiotherapy, occupational therapy, and Cesar/Mensendieck exercise therapy.

## 5 Conclusion

This study demonstrates that people with MS place a high value on physiotherapy, occupational therapy, and posture exercise therapy, which have a lasting impact and are essential from the point of diagnosis. The values and needs of people with MS are dynamic and multifaceted, evolving over time in relation to disease stage and changing life circumstances. Policymakers, health insurers, and healthcare providers should recognize these evolving needs and prioritize patient-centered, integrated, and sustainable allied healthcare for people with MS.

## Data Availability

The raw data supporting the conclusions of this article will be made available by the authors, upon reasonable request.
