# Supplementary material for: Importance of physiotherapy and occupational therapy according to people with multiple sclerosis—results from an online survey
Source: Front Rehabil Sci. 2026 May 12;7:1832561. doi: 10.3389/fresc.2026.1832561 (PMC13201487; doi:10.3389/fresc.2026.1832561)
Supplement: Supplementary file 1 [file Datasheet1.pdf]

## SUPPLEMENTARY TABLES

**Supplementary Table 1.** Comorbidities besides MS that required medical care from a physician (n=193)

| **Comorbidities** | N | % |
|---|---|---|
| No | 112 | 58.0 |
| Yes | 81 | 42.0 |
| | | |
| **Type of comorbidity** | | |
| Cardiovascular | 8 | 4.1 |
| Lungs | 11 | 5.7 |
| Cancer | 6 | 3.1 |
| Eyes | 11 | 5.7 |
| Ears, nose, or throat | 7 | 3.6 |
| Stomach, liver, intestines | 12 | 6.2 |
| Kidney, bladder, prostate, reproductive organs | 19 | 9.8 |
| Musculoskeletal | 15 | 7.8 |
| Skin | 9 | 4.7 |
| Brain or nerves, other than MS | 8 | 4.1 |
| Psychiatric | 5 | 2.6 |
| Hormonal, metabolic, immune system | 10 | 5.2 |
| Other | 21 | 10.9 |

**Supplementary Table 2.** Relationship between perceived burden of MS and the patient-reported outcomes MSIS29, MSWS-12, AMSQ-SF, and ESES.

| Perceived Burden MS | | MSIS29 phys | MSIS29 psy | MSIS29 total | MSWS-12 | AMSQ-SF | ESES |
|---|---|---|---|---|---|---|---|
| No | N | 1 | 1 | 1 | 1 | 1 | 1 |
| | Median | 13.75 | 2.78 | 10.34 | 27.08 | .00 | 16.67 |
| | Minimum | 13.75 | 2.78 | 10.34 | 27.08 | .00 | 16.67 |
| | Maximum | 13.75 | 2.78 | 10.34 | 27.08 | .00 | 16.67 |
| Little | N | 22 | 22 | 22 | 22 | 22 | 22 |
| | Median | 11.25 | 8.33 | 11.21 | 15.63 | .00 | 88.33 |
| | Minimum | 1.25 | .00 | .86 | .00 | .00 | 66.67 |
| | Maximum | 51.25 | 33.33 | 42.24 | 60.42 | 22.00 | 100.00 |
| Moderate | N | 92 | 92 | 92 | 90 | 92 | 87 |
| | Median | 28.75 | 19.44 | 26.72 | 55.21 | 6.00 | 80.00 |
| | Minimum | 3.75 | .00 | 3.45 | 2.08 | .00 | 43.33 |
| | Maximum | 76.25 | 80.56 | 70.69 | 100.00 | 82.00 | 100.00 |
| Much | N | 64 | 64 | 64 | 56 | 64 | 59 |
| | Median | 53.75 | 30.56 | 45.2586 | 76.0417 | 15.00 | 73.33 |
| | Minimum | 13.75 | .00 | 18.97 | 6.25 | .00 | 13.33 |
| | Maximum | 86.25 | 88.89 | 79.31 | 100.00 | 80.00 | 100.00 |
| Very Much | N | 11 | 11 | 11 | 6 | 11 | 10 |
| | Median | 77.50 | 41.67 | 65.52 | 89.58 | 50.00 | 76.67 |
| | Minimum | 40.00 | 11.11 | 37.07 | 10.42 | 4.00 | 16.67 |
| | Maximum | 95.00 | 83.33 | 83.62 | 100.00 | 78.00 | 100.00 |
| Total | N | 190 | 190 | 190 | 175 | 190 | 179 |
| | Median | 36.88 | 22.22 | 33.62 | 60.42 | 8.00 | 80.00 |
| | Minimum | 1.25 | .00 | .86 | .00 | .00 | 13.33 |
| | Maximum | 95.00 | 88.89 | 83.62 | 100.00 | 82.00 | 100.00 |
| Spearman Correlation Coefficient | | .728 | .432 | .715 | .536 | .451 | -.274 |

**Supplementary Table 3**
**Characteristics of current physiotherapy, occupational therapy, and posture exercise therapy among people with MS.**

This table presents detailed information on the current use of physiotherapy (N = 168), occupational therapy (N = 43), and posture exercise therapy (Cesar/Mensendieck; N = 4), including duration of the current therapy episode, frequency of sessions, setting (location and form of therapy), and participants' expectations regarding the continuation or completion of therapy. These data complement the summary description provided in Section 3.2 (Therapy awareness and utilization).

**Table 3A. Characteristics of current physiotherapy (N = 168)**

| Physiotherapy characteristics | N | % |
|---|---|---|
| **Duration of current physiotherapy** | | |
| Less than one month | 3 | 1.8 |
| 1–3 months | 6 | 3.6 |
| 4–6 months | 5 | 3.0 |
| 7–12 months | 5 | 3.0 |
| Longer than one year | 148 | 88.1 |
| I don't know | 1 | 0.6 |
| **Frequency of physiotherapy** | | |
| One session per week | 46 | 27.4 |
| Two sessions per week | 73 | 43.5 |
| More than two sessions per week | 14 | 8.3 |
| One session per month | 2 | 1.2 |
| Two sessions per month | 10 | 6.0 |
| Other | 23 | 13.7 |
| **Location of physiotherapy** | | |
| Physiotherapy practice in my neighborhood | 127 | 75.6 |
| Medical training center or fitness facility | 11 | 6.5 |
| Physiotherapist visits me at home | 9 | 5.4 |
| MS center in a hospital | 0 | 0.0 |
| General hospital | 1 | 0.6 |
| Nursing or residential home | 2 | 1.2 |
| Rehabilitation center | 2 | 1.2 |
| Other | 16 | 9.5 |
| **Form of physiotherapy** | | |
| Individual therapy | 100 | 59.5 |
| Group therapy | 19 | 11.3 |
| Combination of individual and group therapy | 31 | 18.5 |
| Other | 18 | 10.7 |
| **Which situation applies to you?** | | |
| I need physiotherapy for a longer period of time | 145 | 86.3 |
| I will complete physiotherapy after some time | 8 | 4.8 |
| I do not know how long I will need physiotherapy | 15 | 8.9 |

**Table 3B. Characteristics of current occupational therapy (N = 43)**

| Occupational therapy characteristics | N | % |
|---|---|---|
| **Duration of current occupational therapy** | | |
| Less than one month | 7 | 16.3 |
| 1–3 months | 7 | 16.3 |
| 4–6 months | 5 | 11.6 |
| 7–12 months | 7 | 16.3 |
| Longer than one year | 15 | 34.9 |
| I don't know | 2 | 4.7 |
| **Frequency of occupational therapy** | | |
| One session per week | 4 | 9.3 |
| One session per month | 8 | 18.6 |
| Two sessions per month | 5 | 11.6 |
| Once every 3–6 months | 1 | 2.3 |
| 1–2 times per year | 5 | 11.6 |
| As needed | 11 | 25.6 |
| Therapy has been completed | 7 | 16.3 |
| Other | 2 | 4.7 |
| **Location of occupational therapy** | | |
| Occupational therapy practice in my neighborhood | 11 | 26.8 |
| Medical training center or fitness facility | 1 | 2.4 |
| Occupational therapist visits me at home | 13 | 31.7 |
| MS center in a hospital | 3 | 7.3 |
| General hospital | 3 | 7.3 |
| Nursing or residential home | 2 | 4.9 |
| Rehabilitation center | 4 | 9.8 |
| Other | 1 | 2.4 |
| Not applicable | 3 | 7.3 |
| **Form of occupational therapy** | | |
| Individual therapy | 29 | 67.4 |
| Group therapy | 0 | 0.0 |
| Combination of individual and group therapy | 2 | 4.7 |
| Home visits | 2 | 4.7 |
| Advice/thinking along (consultative role) | 3 | 7.3 |
| Not applicable | 6 | 14.0 |
| **Which situation applies to you?** | | |
| I need occupational therapy for a longer period of time | 9 | 20.9 |
| I will complete occupational therapy after some time | 28 | 65.1 |
| I do not know how long I will need occupational therapy | 6 | 14.0 |

**Table 3C. Characteristics of current posture exercise therapy (N = 4)**

| Posture exercise therapy characteristics | N | % |
|---|---|---|
| **Duration of current posture exercise therapy** | | |
| Less than one month | 0 | 0.0 |
| 1–3 months | 0 | 0.0 |
| 4–6 months | 0 | 0.0 |
| 7–12 months | 0 | 0.0 |
| Longer than one year | 4 | 100.0 |
| I don't know | 0 | 0.0 |
| **Frequency of posture exercise therapy** | | |
| One session per week | 2 | 50.0 |
| One session per month | 1 | 25.0 |
| Two sessions per month | 1 | 25.0 |
| Once every 3–6 months | 0 | 0.0 |
| 1–2 times per year | 0 | 0.0 |
| As needed | 0 | 0.0 |
| Other | 0 | 0.0 |
| **Location of posture exercise therapy** | | |
| Posture exercise therapy practice in my neighborhood | 3 | 75.0 |
| Medical training center or fitness facility | 0 | 0.0 |
| Posture exercise therapist visits me at home | 0 | 0.0 |
| MS center in a hospital | 0 | 0.0 |
| General hospital | 0 | 0.0 |
| Nursing or residential home | 0 | 0.0 |
| Rehabilitation center | 0 | 0.0 |
| Other | 1 | 25.0 |
| **Form of posture exercise therapy** | | |
| Individual therapy | 4 | 100.0 |
| Group therapy | 0 | 0.0 |
| Combination of individual and group therapy | 0 | 0.0 |
| Home visits | 0 | 0.0 |
| Advice/thinking along (consultative role) | 0 | 0.0 |
| **Which situation applies to you?** | | |
| I need posture exercise therapy for a longer period of time | 2 | 50.0 |
| I will complete posture exercise therapy after some time | 0 | 0.0 |
| I do not know how long I will need posture exercise therapy | 2 | 50.0 |

**Supplementary Table 4a.** Satisfaction with current physiotherapy (N=168)

| | Very dissatisfied % | Dissatisfied % | Neutral % | Satisfied % | Very satisfied % |
|---|---|---|---|---|---|
| a. The therapy you are receiving or have received in the past 3 months | 0.6 | 1.8 | 4.8 | 28.6 | 64.3 |
| b. Your therapist's knowledge of MS | 1.2 | 3.6 | 13.1 | 34.5 | 47.6 |
| c. How therapy matches your request for therapy | 0.6 | 3.6 | 7.7 | 31.5 | 56.5 |
| d. How therapy fits your personal situation | 0.6 | 3.6 | 5.4 | 29.2 | 61.3 |
| e. The results of the treatment (so far) | 1.2 | 2.4 | 14.3 | 38.1 | 44.0 |
| f. The trust you have in your therapist | 0.6 | 0.6 | 4.8 | 31.0 | 63.1 |
| g. The time available for a therapy appointment | 0.6 | 2.4 | 10.7 | 35.7 | 50.6 |
| h. The contact you have with your therapist | 0.6 | 1.2 | 4.2 | 28.6 | 65.5 |
| i. The input you have in the therapy you receive | 0.6 | 0.6 | 8.3 | 27.4 | 63.1 |
| j. The therapy aligns with your needs and preferences | 0.6 | 2.4 | 8.3 | 29.8 | 58.9 |
| k. Availability of therapy at a time that suits you and as quickly as you want | 1.2 | 0.6 | 14.3 | 29.8 | 54.2 |
| l. How your therapist collaborates with other healthcare providers | 1.8 | 3.6 | 29.8 | 31.0 | 33.0 |
| m. The accessibility of the therapy practice | 0.6 | 0.6 | 7.7 | 36.9 | 54.2 |
| n. The amount you have to pay for the therapy yourself | 3.0 | 5.4 | 28.0 | 21.4 | 42.3 |

**Supplementary Table 4b.** Satisfaction with current occupational therapy (N=43)

| | Very dissatisfied % | Dissatisfied % | Neutral % | Satisfied % | Very satisfied % |
|---|---|---|---|---|---|
| a. The therapy you are receiving or have received in the past 3 months | | | 7.0 | 39.5 | 53.5 |
| b. Your therapist's knowledge of MS | | | 14.0 | 34.9 | 51.2 |
| c. How therapy matches your request for therapy | | | 9.3 | 39.5 | 51.2 |
| d. How therapy fits your personal situation | | | 7.0 | 48.8 | 44.2 |
| e. The results of the treatment (so far) | | | 16.3 | 46.5 | 37.2 |
| f. The trust you have in your therapist | | | 11.6 | 32.6 | 55.8 |
| g. The time available for a therapy appointment | | | 7.0 | 51.2 | 41.9 |
| h. The contact you have with your therapist | | 4.7 | 2.3 | 34.9 | 58.1 |
| i. The input you have in the therapy you receive | | | 9.3 | 37.2 | 53.5 |
| j. The therapy aligns with your needs and preferences | | 2.3 | 9.3 | 39.5 | 48.8 |
| k. Availability of therapy at a time that suits you and as quickly as you want | | 4.7 | 9.3 | 39.5 | 46.5 |
| l. How your therapist collaborates with other healthcare providers | | 4.7 | 16.3 | 37.2 | 41.9 |
| m. The accessibility of the therapy practice | | 2.3 | 14.0 | 39.5 | 44.2 |
| n. The amount you have to pay for the therapy yourself | | 4.7 | 44.2 | 18.6 | 32.6 |

**Supplementary Table 4c.** Satisfaction with current posture exercise therapy (N=4)

| | Very dissatisfied % | Dissatisfied % | Neutral % | Satisfied % | Very satisfied % |
|---|---|---|---|---|---|
| a. The therapy you are receiving or have received in the past 3 months | | | | 25.0 | 75.0 |
| b. Your therapist's knowledge of MS | | | | 25.0 | 75.0 |
| c. How therapy matches your request for therapy | | | | 25.0 | 75.0 |
| d. How therapy fits your personal situation | | | | 25.0 | 75.0 |
| e. The results of the treatment (so far) | | | | 50.0 | 50.0 |
| f. The trust you have in your therapist | | | | 25.0 | 75.0 |
| g. The time available for a therapy appointment | | | | 25.0 | 75.0 |
| h. The contact you have with your therapist | | | | 25.0 | 75.0 |
| i. The input you have in the therapy you receive | | | | 25.0 | 75.0 |
| j. The therapy aligns with your needs and preferences | | | | 25.0 | 75.0 |
| k. Availability of therapy at a time that suits you and as quickly as you want | | | | 25.0 | 75.0 |
| l. How your therapist collaborates with other healthcare providers | | | | 50.0 | 50.0 |
| m. The accessibility of the therapy practice | | | | 25.0 | 75.0 |
| n. The amount you have to pay for the therapy yourself | | | | 25.0 | 75.0 |
